# Supplementary material for: Burden of mortality and its predictors among TB-HIV co-infected patients in Ethiopia: Systematic review and meta-analysis
Source: PLoS One. 2024 Nov 7;19(11):e0312698. doi: 10.1371/journal.pone.0312698 (PMC11542784; doi:10.1371/journal.pone.0312698)
Supplement: S2 Checklist — (DOCX) [file pone.0312698.s002.docx]

# JBI critical appraisal checklist for cohort studies

| **Primary studies** |  | **JBI’s critical appraisal questions** | | | | | | | | | | Overall quality score (%) | Included |
| --- | --- | --- | --- | --- | --- | --- | --- | --- | --- | --- | --- | --- | --- |
|  | Q1 | Q2 | Q3 | Q4 | Q5 | Q6 | Q7 | Q8 | Q9 | Q10 | Q11 |  |  |
| Atalel et al.,2018 | Y | Y | Y | Y | N | Y | Y | y | N | Y | Y | 90.9 | √ |
| Birhan et al.,2021 | Y | Y | Y | Y | N | Y | Y | Y | N | Y | Y | 82 | √ |
| Chanie et al., 2021 | y | Y | Y | Y | N | Y | Y | N | N | Y | Y | 82 | √ |
| Dawit et al.,2021 | Y | Y | Y | Y | N | Y | Y | Y | Y | Y | Y | 90.9 | √ |
| Gemechu et al.,2022 | Y | Y | Y | Y | N | Y | Y | Y | Y | N | Y | 90.9 | √ |
| Gesesew et al.,2016 | Y | Y | Y | Y | N | Y | Y | Y | N | Y | Y | 82 | √ |
| Gezea eta l.,2020 | Y | Y | Y | Y | N | Y | Y | Y | N | Y | Y | 82 | √ |
| Habtegiorgis et al.,2023 | Y | Y | Y | Y | Y | Y | Y | Y | N | N | Y | 82 | √ |
| Lelisho et al.,2022 | Y | Y | Y | Y | N | Y | Y | Y | Y | N | Y | 82 | √ |
| Nigussie et al.,2021 | Y | Y | Y | Y | N | Y | Y | Y | N | Y | Y | 82 | √ |
| Refera et al.,2013 | Y | Y | Y | Y | N | Y | Y | Y | N | N | Y | 82 | √ |
| Silesh et al.,2013 | Y | Y | Y | Y | N | Y | Y | Y | N | Y | Y | 82 | √ |
| Sime et al.,2022 | Y | Y | Y | Y | N | Y | Y | Y | N | N | Y | 82 | √ |
| Teklu et al.,2017 | Y | Y | Y | Y | N | Y | Y | Y | N | Y | Y | 82 | √ |
| Wondimu et al.,2020 | Y | Y | Y | Y | N | Y | Y | Y | N | N | Y | 82 | √ |

Q1: Were the two groups similar and recruited from the same population?

Q2: Were the exposures measured similarly to assign people to both exposed and unexposed (Marie et al., 2022)groups?

Q3: Was the exposure measured in a valid and reliable way?

Q4: Were confounding factors identified?

Q5: Were strategies to deal with confounding factors stated?

Q6: Were the groups/participants free of the outcome at the start of the study (or at the moment of exposure)?

Q7: Were the outcomes measured in a valid and reliable way?

Q8: Was the follow up time reported and sufficient to be long enough for outcomes to occur?

Q9: Was follow up complete, and if not, were the reasons to loss to follow up described and explored?

Q10: Were strategies to address incomplete follow up utilized?

Q11: Was appropriate statistical analysis used?

**NB**: Y: Yes, N: No, U: Unclear, Q: Question. The overall score is calculated by counting the number of Y’s in each row.
