# Supplementary material for: Burden of mortality and its predictors among TB-HIV co-infected patients in Ethiopia: Systematic review and meta-analysis
Source: PLoS One. 2024 Nov 7;19(11):e0312698. doi: 10.1371/journal.pone.0312698 (PMC11542784; doi:10.1371/journal.pone.0312698)
Supplement: S1 Table — (DOCX) [file pone.0312698.s004.docx]

| Search in each key words and Mesh terms | **Pumed/MEDLINE database** |
| --- | --- |
| **#1** | burden OR magnitude |
| **#2** | Mortality OR survival OR "Survival"[Mesh] OR "Mortality"[Mesh] |
| **#3** | Predictors OR “associated factors” OR determinants OR “risk factors” |
| **#4** | “associated factors” OR determinants OR “risk factors” |
| **#5** | “Tuberculosis-Human Immunodeficiency Virus co- infection” OR “TB-HIV co-infection” OR “TB-HIV co-infected patients” OR “TB-HIV co-infected persons” OR “TB-HIV co-infected adolescents” |
|  |  |
| **Final** | (((("Incidence"[Mesh] OR proportions[tw] OR "incidence rate" OR "incidence density" OR "time to death" [tw] OR burden[tw] OR magnitude[tw] AND ((ffrft[Filter]) AND (humans[Filter]) AND (English[Filter]))) AND (mortality[tw] OR survival[tw] OR "Survival"[Mesh] OR "Mortality"[Mesh] AND ((ffrft[Filter]) AND (humans[Filter]) AND (English[Filter])))) AND (predictors[tw] OR "associated factors" OR determinants OR "risk factors" AND ((ffrft[Filter]) AND (humans[Filter]) AND (English[Filter])))) AND ("Tuberculosis-Human Immunodeficiency Virus co- infection" OR "TB-HIV co-infection" OR "TB-HIV co-infected patients" OR "TB-HIV co-infected persons" OR "TB-HIV co-infected adolescents" AND ((ffrft[Filter]) AND (humans[Filter]) AND (English[Filter])))) AND (Ethiopia AND ((ffrft[Filter]) AND (humans[Filter]) AND (English[Filter]))) AND ((ffrft[Filter]) AND (humans[Filter]) AND (English[Filter])) |
|  |  |

S1 Table: search strategies and entry terms from electronic data bases on mortality and its Predictors among TB-HIV co-infected patients in Ethiopia: systematic and meta-analysis
