# Supplementary material for: Burden of mortality and its predictors among TB-HIV co-infected patients in Ethiopia: Systematic review and meta-analysis
Source: PLoS One. 2024 Nov 7;19(11):e0312698. doi: 10.1371/journal.pone.0312698 (PMC11542784; doi:10.1371/journal.pone.0312698)
Supplement: S2 Table — (DOCX) [file pone.0312698.s005.docx]

**Table**: A list of excluded studies on a study of burden of mortality and its Predictors among TB-HIV co-infected patients in Ethiopia: systematic review and Meta-analysis.

| **First authors/publication year** | **Reason of exclusion** |
| --- | --- |
| 1. (Asgedom et al., 2018b) | Title, study design, outcome |
| 1. (Welekidan et al., 2020) | Title, study design, outcome |
| 1. (Birlie et al., 2015) | Title, outcome |
| 1. (Sime et al., 2022) | Title, outcome |
| 1. (Zohar et al., 2014) | Study setting |
| 1. (Workie et al., 2021a) | Study only on HIV |
| 1. (Zemariam et al., 2024b) | Title and Abstract |
| 1. (Misgina et al., 2019) | Title and Abstract |
| 1. (Temesgen et al., 2019) | Study only on HIV |
| 1. (Biset Ayalew, 2017) | Title and Abstract |
| 1. (Kassa et al., 2019a) | Title and Abstract |
| 1. (Gezae et al., 2019) | Title and Abstract |
| 1. (Woldeyohannes et al., 2021) | Title and Abstract |
| 1. (Admasu et al., 2024) | Title and outcome |
| 1. (Girma et al., 2024a) | Title and outcome |
| 1. (Nagu et al., 2017b) | Study area and outcome |
| 1. (Alemu et al., 2024) | Study area and outcome |
| 1. (Sisay et al., 2018) | Title and outcome |
| 1. (Desta et al., 2021) | Title and outcome |
| 1. (Kassa et al., 2024) | Title and Abstract |
| 1. (Woldegeorgis et al., 2022) | Title and Abstract |
| 1. (Ayele et al., 2015) | Title and Abstract |
| 1. (Geremew et al., 2019) | Title and Abstract |
| 1. (Akolo et al., 2010) | Study area |
| 1. (Sabasaba et al., 2019) | Study area |
| 1. (Anulo et al., 2024) | Title and Abstract |
| 1. (Gezae et al., 2023) | Title and Abstract |
| 1. (Baldeh et al., 2023) | Study area |
| 1. (Zemariam et al., 2024a) | Title and Abstract |
| 1. (Gebremariam et al., 2016) | Title and Abstract |
| 1. (Assefa et al., 2014b) | Title and Abstract |
| 1. (Anlay et al., 2016) | Title and Abstract |
| 1. (Assebe et al., 2015a) | Title and Abstract |
| 1. (Burke et al., 2021) | Title |
| 1. (Dodd et al., 2017) | Title |
| 1. (Getaneh et al., 2023) | Title and Abstract |
| 1. (Bogale et al., 2022) | Title and methods |
| 1. (Kiros et al., 2022) | Title and Abstract |
| 1. (Getaneh et al., 2022b) | Title |
| 1. (Belay et al., 2021) | Title and Abstract |
| 1. (Haile et al., 2016) | Title and Abstract |
| 1. (Sonya et al.,2013) | Title and Abstract |
| 1. (Lalem et al.,2013) | Title and Abstract |
| 1. (Tolla et al.,2013) | Title and Abstract |
| 1. (Negash et al.,2013) | Title and Abstract |
| 1. (Abrha et al., 2015) | Title and Abstract |
| 1. (Kegne et al., 2024) | Title and Abstract |
| 1. (Birhan et al., 2021) | Title and Abstract |
| 1. (Fite et al., 2019) | Title and Abstract |
| 1. (Lenjisa et al., 2015) | Title and Abstract |
| 1. (Gebreyes, 2022) | Title and Abstract |
| 1. (Tola et al., 2019) | Title |
| 1. (Kassa et al., 2020) | Title |
| 1. (Seyoum et al., 2022) | Title and outcome |
| 1. (Asgedom et al., 2018a) | Title and outcome |
| 1. (Mitku et al., 2016) | Title |
| 1. (Workie et al., 2021b) | Title |
| 1. (Teshome Kefale and Anagaw, 2017) | Title |
| 1. (Birhan et al., 2022) | Title and Abstract |
| 1. (Assefa et al., 2014b) | Title and Abstract |
| 1. (Anlay et al., 2016) | Title and Abstract |
| 1. (Assebe et al., 2015a) | Title and Abstract |
| 1. (Burke et al., 2021) | Title |
| 1. (Hailay Abrha et al., 2015) | Title and Abstract |
| 1. (Asgedom et al., 2018a) | Title and Abstract |
| 1. (Birhan et al., 2022) | Title |
| 1. (Sinshaw et al., 2017) | Title and Abstract |
| 1. (Fiseha et al., 2015) | Title and Abstract |
| 1. (Esmael et al., 2013) | Title and Abstract |
| 1. (Asebe et al., 2015) | Title |
| 1. (Tanue et al., 2019) | Study setting |
| 1. (Ali et al., 2016) | Title and Abstract |
| 1. (Tarekegne et al., 2016) | Title and Abstract |
| 1. (Mekonnen et al., 2015) | Title |
| 1. (Teweldemedhin et al., 2018) | Title and Abstract |
| 1. (Feleke et al., 2020) | Title and Abstract |
| 1. (Ayana et al., 2021) | Title and Abstract |
| 1. (Getaw and Tigu, 2024) | Title and Abstract |
| 1. (Shimbre et al., 2020) | Title and Abstract |
| 1. (Belew et al., 2020) | Title and Abstract |
| 1. (Woldeamanuel and Mingude, 2018) | Title and Abstract |
| 1. (Eticha and Kassa, 2014) | Title and Abstract |
| 1. (Jabir et al., 2022b) | Title and Abstract |
| 1. (Getaneh et al., 2022a) | Title and Abstract |
| 1. (Fekadu et al., 2020) | Title and Abstract |
| 1. (Tesfaye et al., 2018) | Title and Abstract |
| 1. (Seid et al., 2023) | Title and Abstract |
| 1. (Teketelew et al., 2022) | Title and Abstract |
| 1. (Muhie, 2024) | Title and Abstract |
| 1. (Zeleke et al., 2014) | Title and Abstract |
| 1. (Bekalo et al., 2017) | Title and Abstract |
| 1. (Edessa et al., 2021) | Title and Abstract |
| 1. (Gemechu and Debusho, 2023) | Title and Abstract |
| 1. (Tola et al., 2021) | Title and Abstract |
| 1. (Ahmed et al., 2015) | Title and Abstract |
| 1. (Teshale et al., 2021) | Title and Abstract |
| 1. (Sorsa and Kaso, 2021) | Title and Abstract |
| 1. (Ahmed et al., 2018) | Title and Abstract |
| 1. (Bayabil and Seyoum, 2021) | Title and Abstract |
| 1. (BEYIKRTA, 2022) | Title and Abstract |
| 1. (Kiros et al., 2020) | Title and Abstract |
| 1. (Abongomera et al., 2017) | Title and Abstract |
| 1. (Beyene et al., 2016) | Title and Abstract |
| 1. (Dalbo and Tamiso, 2016) | Study area |
| 1. (Getu et al., 2022) | Title and Abstract |
| 1. (Debelu, 2022) | Title and Abstract |
| 1. (Shaik et al., 2022) | Study area |
| 1. (Momenyan et al., 2023) | Study area |
| 1. (Abdullahi et al., 2023) | Study area |
| 1. (Getachew et al., 2019) | Title and Abstract |
| 1. (Mekebo et al., 2020) | Title and Abstract |
| 1. (Argawu, 2020) | Title and Abstract |
| 1. (Meseret et al., 2017) | Title and Abstract |
| 1. (Alemu et al., 2020) | Title and Abstract |
| 1. (Diriba and Awulachew, 2022) | Title and Abstract |
| 1. (Ausman Ahmed et al., 2018) | Title and Abstract |
| 1. (Mera et al., 2020) | Title and Abstract |
| 1. (Ahmed et al., 2018) | Title and Abstract |
| 1. (Ahmed et al., 2015) | Title and Abstract |
| 1. (Alemie and Gebreselassie, 2014) | Title and Abstract |
| 1. (Belay et al., 2013) | Title and Abstract |
| 1. (Melkamu et al., 2013) | Title and Abstract |
| 1. (Adane et al., 2020) | Title and Abstract |
| 1. (Gisso et al., 2022) | Title and Abstract |
| 1. (Geremew et al., 2022) | Title and Abstract |
| 1. (Shimeles et al., 2019) | Title and Abstract |
| 1. (Hailu et al., 2020) | Title and Abstract |
| 1. (Hassen Ali et al., 2013) | Title and Abstract |
| 1. (Atey et al., 2020) | Title and Abstract |
| 1. (Reepalu et al., 2016) | Title and Abstract |
| 1. (Sime et al., 2022, Kebede et al., 2021c) | Title and Abstract |
| 1. Kebede et al., 2021) | Title and Abstract |
| 1. (Deribew et al., 2010) | Title and Abstract |
| 1. (Temitayo-Oboh et al., 2022b) | Study area |
| 1. (Amare, 2015) | Title and Abstract |
| 1. (Hailu et al., 2020) | Title and Abstract |
| 1. (Hassen Ali et al., 2013) | Title and Abstract |
| 1. (Deribew et al., 2010) | Title and Abstract |
| 1. (Temitayo-Oboh et al., 2022b) | Title and Abstract |
| 1. (Getahun et al., 2023) | Title and Abstract |
| 1. (Kassa et al., 2019b) | Title and Abstract |
| 1. (Assebe et al., 2020) | Title and Abstract |
| 1. (Jabir et al., 2022a) | Title and Abstract |
| 1. (Bayowa et al., 2023) | Title and Abstract |
| 1. (Deribew et al., 2009) | Title and Abstract |
| 1. (Addis Alene et al., 2013) | Title and Abstract |
| 1. (Deribew et al., 2013) | Title and Abstract |
| 1. (Zenbaba et al., 2022) | Title and Abstract |
| 1. (Tadiwos) | Title and Abstract |
| 1. (Fantaw et al., 2018) | Title and Abstract |
| 1. (Yakob et al., 2018) | Title and Abstract |
| 1. (Simieneh et al., 2017) | Title and Abstract |
| 1. (Abdu et al., 2021) | Title and Abstract |
| 1. (Aung et al., 2019) | Study area |
| 1. (Adegeh and Kebede, 2021) | Title and Abstract |
| 1. (Girma et al., 2024b) | Title and Abstract |
| 1. (Mohammed and Gebremariam, 2015) | Title and Abstract |
| 1. (Megersa, 2013) | Title and Abstract |
| 1. (Alemu et al., 2021) | Title and Abstract |
| 1. (Gebre-Mariam, 2009) | Title and Abstract |
| 1. (Mollel et al., 2020) | Study area |
| 1. (Wakjira et al., 2022) | Title and Abstract |
| 1. (Geleso, 2020) | Title and Abstract |
| 1. (Beshaw et al., 2021) | Title and Abstract |
| 1. (Rocha et al., 2021) | Title and Abstract |
| 1. (Azeez et al., 2018) | Study area |
| 1. (Abay et al., 2015) | Title and Abstract |
| 1. (Shaweno and Worku, 2012) | Title and Abstract |
| 1. (Desalegn) | Title and Abstract |
| 1. (Ketema et al., 2019) | Title and Abstract |
| 1. (Kazemian et al., 2024) | Study area |
| 1. (Kassa et al., 2012a) | Title and Abstract |
| 1. (Osei et al., 2020) | Study area |
| 1. (Mugusi et al., 2009) | Title and Abstract |
| 1. (Mama et al., 2018) | Title and Abstract |
| 1. (Umeta et al., 2022) | Title and Abstract |
| 1. (Rossetto et al., 2019) | Title and Abstract |
| 1. (Worku et al., 2018) | Title and Abstract |
| 1. (Agbor et al., 2015) | Study setting |
| 1. (Asuke et al., 2020) | Study setting |
| 1. (Bizuneh et al., 2024b) | Title and Abstract |
| 1. (Tadesse et al., 2022) | Title and Abstract |
| 1. (Tegegne and Minwagaw, 2022) | Title and Abstract |
| 1. (Adejumoa et al., 2017) | Study setting |
| 1. (Ambaye and Tsegaye, 2021) | Title and Abstract |
| 1. (Omara, 2022) | Study setting |
| 1. (Abdilahi et al., 2024) | Title and Abstract |
| 1. (Debash et al., 2023) | Title and Abstract |
| 1. (Tarekegne et al., 2016) | Title and Abstract |
| 1. (Gebreegziabher et al., 2016) | Title and Abstract |
| 1. (Wondmagegn et al., 2020) | Title and Abstract |
| 1. (Shah et al., 2021) | Study setting |
| 1. (Getachew et al., 2023) | Title and Abstract |
| 1. (Terefe and Gebrewold, 2018) | Title and Abstract |
| 1. (Argaw et al., 2023) | Title and Abstract |
| 1. (Eyasu et al., 2014) | Title and Abstract |
| 1. (Negash et al., 2019) | Title and Abstract |
| 1. (Assefa et al., 2014a) | Title and Abstract |
| 1. (Gebremicael et al., 2021) | Title and Abstract |
| 1. (Goh Chun Chao and Avoi, 2023) | Study setting |
| 1. (Nguyen et al., 2018) | Study setting |
| 1. (Kajogoo et al., 2022) | Study setting |
| 1. (Dires et al., 2021) | Title and Abstract |
| 1. (Abedi et al., 2019) | Title and Abstract |
| 1. (Melkamu et al., 2013) | Title and Abstract |
| 1. (Jemal et al., 2015) | Title and Abstract |
| 1. (AHMED, 2022) | Title and Abstract |
| 1. (Abebe and Angamo, 2015) | Title and Abstract |
| 1. (Wang et al., 2024) | Setting |
| 1. (Degu, 2015) | Title and Abstract |
| 1. (Abebe et al., 2014) | Title and Abstract |
| 1. (Kibret et al., 2013) | Title and Abstract |
| 1. (Bristedt et al., 2024) | Title and Abstract |
| 1. (Girum et al., 2020) | Title and Abstract |
| 1. (Nugus and Irena, 2020) | Title and Abstract |
| 1. (Abdu et al., 2021) | Title and Abstract |
| 1. (Lawn et al., 2005) | Study setting |
| 1. (Asefa et al., 2024) | Title and Abstract |
| 1. (Aemro et al., 2020) | Title and Abstract |
| 1. (Azanaw et al., 2021a) | Title and Abstract |
| 1. (Mebratu et al., 2022) | Title and Abstract |
| 1. (Jones et al., 2000) | Title and Abstract |
| 1. (Sime et al., 2022) | Title and Abstract |
| 1. (Bizuneh et al., 2024a) | Title and Abstract |
| 1. (Kassa et al., 2012b) | Title and Abstract |
| 1. (Moreno et al., 2008) | Title and Abstract |
| 1. (Jerene et al., 2006) | Title and Abstract |
| 1. (Kiros et al., 2020) | Title and Abstract |
| 1. (Yirdaw et al., 2019) | Title and Abstract |
| 1. (Pathmanathan et al., 2017) | Title and Abstract |
| 1. (Mupfumi et al., 2019) | Title and Abstract |
| 1. (Ausman Ahmed et al., 2018) | Title and Abstract |
| 1. (Tegegnework et al., 2024) | Title and Abstract |
| 1. (Ganesan et al., 2023) | Title and Abstract |
| 1. (Ganesan et al., 2023) | Title and Abstract |
| 1. (Adugna et al., 2020) | Title and Abstract |
| 1. (Ismail, 2014) | Title and Abstract |
| 1. (Mekonnen et al., 2016) | Title and Abstract |
| 1. (Ayelign et al., 2020) | Title and Abstract |
| 1. (Mwatenga et al., 2024) | Title and Abstract |
| 1. (HAWULET, 2021) | Title and Abstract |
| 1. (Ermeko et al., 2021) | Title and Abstract |
| 1. (Ogyiri et al., 2019) | Title and Abstract |
| 1. (Agegnehu et al., 2022) | Title and Abstract |
| 1. (Teferi et al., 2021) | Title and Abstract |
| 1. (Dale et al., 2017) | Title and Abstract |
| 1. (Kassu et al., 2007) | Title and Abstract |
| 1. (Abdulkader et al., 2019) | Title and Abstract |
| 1. (Mekonnen, 2021) | Title and Abstract |
| 1. (Burusie et al., 2023) | Title and Abstract |
| 1. (Taye et al., 2018) | Title and Abstract |
| 1. (Obeagu and Onuoha, 2023) | Title and Abstract |
| 1. (Esmael et al., 2013) | Title and Abstract |
| 1. (Kadia et al., 2020) | Title and Abstract |
| 1. (Muhie and Tegegne, 2024) | Title and Abstract |
| 1. (Anye et al., 2020) | Title and Abstract |
| 1. (Balkissou et al., 2022) | Title and Abstract |
| 1. (Kassa et al., 2016) | Title and Abstract |
| 1. (Tshitenge et al., 2018) | Title and Abstract |
| 1. (Tanue et al., 2019) | Title and Abstract |
| 1. (Lugutuah et al., 2024) | Title and Abstract |
| 1. (Wotale et al., 2024) | Title and Abstract |
| 1. (Kassaw et al., 2020) | Title and Abstract |
| 1. (Yang et al., 2022) | Title and Abstract |
| 1. (Zenebe et al., 2016) | Title and Abstract |
| 1. (Assebe et al., 2015b) | Title and Abstract |
| 1. (Otiende et al., 2019) | Title and Abstract |
| 1. (Otwombe et al., 2013) | Title and Abstract |
| 1. (Hosu et al., 2024) | Title and Abstract |
| 1. (Suara and Aryee, 2018) | Title and Abstract |
| 1. (Costa et al., 2023) | Title and Abstract |
| 1. (Azanaw et al., 2021b) | Title and Abstract |
| 1. (Tesfaye et al., 2018) | Title and Abstract |
| 1. (Kay et al., 2022) | Title and Abstract |
| 1. (Mukuku et al., 2019) | Title and Abstract |
| 1. (Dodd et al., 2014) | Title and Abstract |
| 1. (Carvalho and Kritski, 2022) | Title and Abstract |
| 1. (Vonasek et al., 2022) | Title and Abstract |
| 1. (Assefa et al., 2022) | Title and Abstract |
| 1. (Seddon and Shingadia, 2014) | Title and Abstract |
| 1. (Jaganath et al., 2022) | Title and Abstract |
| 1. (Snow et al., 2018) | Title and Abstract |
| 1. (Mandalakas et al., 2020) | Title and Abstract |
| 1. (Burusie et al., 2023) | Title and Abstract |
| 1. (Birhanu et al., 2024) | Title and Abstract |
| 1. (Abebaw et al., 2023) | Title and Abstract |
| 1. (Wondmeneh and Mekonnen, 2023) | Title and Abstract |
| 1. (Teweldemedhin et al., 2018) | Title and Abstract |
| 1. (Caminero and Scardigli, 2016) | Title and Abstract |
| 1. (Verkuijl et al.) | Title and Abstract |
| 1. (Suara and Aryee, 2018) | Title and Abstract |
| 1. (Kebede et al., 2021a) | Title and Abstract |
| 1. (Tekese et al., 2023) | Title and Abstract |
| 1. (Alemu et al., 2016) | Title and Abstract |
| 1. (Kebede et al., 2022) | Title and Abstract |
| 1. (Ayalaw et al., 2015a) | Title and Abstract |
| 1. (Jerene et al., 2017) | Title and Abstract |
| 1. (Tsegaye et al., 2023b) | Title and Abstract |
| 1. (Wondifraw et al., 2022) | Title and Abstract |
| 1. (Beshir et al., 2019) | Title and Abstract |
| 1. (Endalamaw et al., 2018) | Title and Abstract |
| 1. (Arage et al., 2019) | Title and Abstract |
| 1. (Braitstein et al., 2006) | Title and Abstract |
| 1. (Biyazin et al., 2022) | Title and Abstract |
| 1. (Bitew et al., 2017) | Title and Abstract |
| 1. (Adem et al., 2014) | Title and Abstract |
| 1. (Alebel et al., 2018) | Title and Abstract |
| 1. (Molla et al., 2022) | Title and Abstract |
| 1. (Taye et al., 2010) | Title and Abstract |
| 1. (Alebel et al., 2020b) | Title and Abstract |
| 1. (Oumer et al., 2019) | Title and Abstract |
| 1. (Marie et al., 2022) | Title and Abstract |
| 1. (Ebissa et al., 2015) | Title and Abstract |
| 1. (Bitew, 2014) | Title and Abstract |
| 1. (Belachew, 2019) | Title and Abstract |
| 1. (Ashagre, 2012) | Title and Abstract |
| 1. (Wubneh and Belay, 2020) | Title and Abstract |
| 1. (Fergusson and Tomkins, 2009) | Title and Abstract |
| 1. (Colecraft, 2008) | Title and Abstract |
| 1. (Sutcliffe et al., 2011) | Title and Abstract |
| 1. (Edmonds et al., 2011) | Title and Abstract |
| 1. (Sashindran and Thakur, 2020) | Title and Abstract |
| 1. (Thimmapuram et al., 2019) | Title and Abstract |
| 1. (Njuguna et al., 2018) | Title and Abstract |
| 1. (Ford et al., 2015) | Title and Abstract |
| 1. (Mwiru et al., 2015) | Title and Abstract |
| 1. (Preidis et al., 2011) | Title and Abstract |
| 1. (Turck et al., 2013) | Title and Abstract |
| 1. (Tsegaye et al., 2023a) | Title and Abstract |
| 1. (Liu et al., 2011) | Title and Abstract |
| 1. (Peters et al., 2019) | Title and Abstract |
| 1. (Ajari and Adewale, 2021) | Title and Abstract |
| 1. (Ndirangu et al., 2011) | Title and Abstract |
| 1. (Frigati et al., 2018) | Title and Abstract |
| 1. (Duggal et al., 2012) | Title and Abstract |
| 1. (Ahmed and Lemma, 2019) | Title and Abstract |
| 1. (Jesson et al., 2015) | Title and Abstract |
| 1. (Wamalwa et al., 2010) | Title and Abstract |
| 1. (Alebel et al., 2021) | Title and Abstract |
| 1. (Rose et al., 2014a) | Title and Abstract |
| 1. (Scrimshaw and SanGiovanni, 1997) | Title and Abstract |
| 1. (Fabusoro and Mejia, 2021) | Title and Abstract |
| 1. (WHO:) | Title and Abstract |
| 1. (USAID) | Title and Abstract |
| 1. (UNICEF., 2021,) | Title and Abstract |
| 1. (UNAIDS:, 2022 Fact sheet ) | Title and Abstract |
| 1. (FHAPCO, 2021-2025, Aynalem et al., 2020) | Title and Abstract |
| 1. (Okeke, 2020) | Title and Abstract |
| 1. (Gondar, 2015) | Title and Abstract |
| 1. (Gibson, 2021) | Title and Abstract |
| 1. (Phafane et al., 2024) | Title and Abstract |
| 1. (Mrema et al., 2024) | Title and Abstract |
| 1. (Mesfin et al., 2012) | Title and Abstract |
| 1. (Gupta et al., 2023) | Title and Abstract |
| 1. (Tiruneh and Deyas, 2020) | Title and Abstract |
| 1. (Galgallo, 2022) | Title and Abstract |
| 1. (Tegbaru Erkyhun, 2007) | Title and Abstract |
| 1. (Tavares et al., 2017) | Title and Abstract |
| 1. (Mollalign et al., 2022) | Title and Abstract |
| 1. (Girum et al., 2018) | Title and Abstract |
| 1. (Kidanemariam, 2014) | Title and Abstract |
| 1. (van Griensven et al., 2018) | Title and Abstract |
| 1. (Dirie et al., 2022) | Title and Abstract |
| 1. (Kebede et al., 2021d) | Title and Abstract |
| 1. (Sade, 2013) | Title and Abstract |
| 1. (Naidoo et al., 2018) | Title and Abstract |
| 1. (Ruseesa et al., 2023) | Title and Abstract |
| 1. (Malede et al., 2015) | Title and Abstract |
| 1. (Andargie et al., 2021) | Title and Abstract |
| 1. (Assefa et al., 2021) | Title and Abstract |
| 1. (Wedajo et al., 2022) | Title and Abstract |
| 1. (Olowookere et al., 2023) | Title and Abstract |
| 1. (Ayele and Amogne, 2021) | Title and Abstract |
| 1. (Kitiabi and Cranmer, 2020) | Title and Abstract |
| 1. (Hu et al., 2022) | Title and Abstract |
| 1. (Amirkhani et al., 2021) | Title and Abstract |
| 1. (Mawuta, 2017) | Title and Abstract |
| 1. (Oyefabi et al., 2023) | Title and Abstract |
| 1. (Afferu et al., 2020) | Title and Abstract |
| 1. (Mugusi et al., 2020) | Title and Abstract |
| 1. (Shilongo, 2022) | Title and Abstract |
| 1. (Muluye et al., 2018) | Title and Abstract |
| 1. (Gyamfi–Gyimah, 2019) | Title and Abstract |
| 1. (Ahmed et al., 2024) | Title and Abstract |
| 1. (Dagnaw et al.) | Title and Abstract |
| 1. (Beza et al., 2013) | Title and Abstract |
| 1. (Bajehson et al., 2019) | Title and Abstract |
| 1. (Gelaw et al., 2019) | Title and Abstract |
| 1. (Magomere and Obwoge, 2018) | Title and Abstract |
| 1. (Ifebunandu et al., 2012) | Title and Abstract |
| 1. (Babawo et al., 2020) | Title and Abstract |
| 1. (Liao et al., 2024) | Title and Abstract |
| 1. (Ayana et al., 2019) | Title and Abstract |
| 1. (Hamada et al., 2021) | Title and Abstract |
| 1. (Hailu et al., 2014) | Title and Abstract |
| 1. (Nogueira et al., 2018) | Title and Abstract |
| 1. (Shaweno et al., 2019) | Title and Abstract |
| 1. (Ochonye et al., 2024) | Title and Abstract |
| 1. (Hamusse, 2017) | Title and Abstract |
| 1. (Chewe et al., 2023) | Title and Abstract |
| 1. (Toru et al., 2022) | Title and Abstract |
| 1. (Bukundi, 2020) | Title and Abstract |
| 1. (Mengesha et al., 2021) | Title and Abstract |
| 1. (Wolde et al., 2023) | Title and Abstract |
| 1. (Lonsako, 2017) | Title and Abstract |
| 1. (Reepalu et al., 2017) | Title and Abstract |
| 1. (Tsegaye et al., 2023c) | Title and Abstract |
| 1. (Agyemang, 2022) | Title and Abstract |
| 1. (Naidoo et al., 2022) | Title and Abstract |
| 1. (Mengesha et al., 2022) | Title and Abstract |
| 1. (Kebede et al., 2021b) | Title and Abstract |
| 1. (Duru et al., 2014) | Title and Abstract |
| 1. (Shamu et al., 2019) | Title and Abstract |
| 1. (Van Hout and Hope, 2019) | Title and Abstract |
| 1. (Bruchfeld et al., 2002) | Title and Abstract |
| 1. (Ifa, 2018) | Title and Abstract |
| 1. (Hailu et al., 2015) | Title and Abstract |
| 1. (Bengura and Managa, 2020) | Title and Abstract |
| 1. (Kaso et al., 2020) | Title and Abstract |
| 1. (Mohammed et al., 2018) | Title and Abstract |
| 1. (Timilsina and Regmi, 2014) | Title and Abstract |
| 1. (Wagnew et al., 2024) | Title and Abstract |
| 1. (Adamu et al., 2017) | Title and Abstract |
| 1. (Limenh et al., 2024) | Title and Abstract |
| 1. (Dembelu et al., 2021) | Title and Abstract |
| 1. (Meressa et al., 2015) | Title and Abstract |
| 1. (Kassa et al., 2013) | Title and Abstract |
| 1. (Bukundi et al., 2021) | Title and Abstract |
| 1. (Ofoegbu and Odume, 2015) | Title and Abstract |
| 1. (Ejeta et al., 2015) | Title and Abstract |
| 1. (IIiyasu et al., 2016) | Title and Abstract |
| 1. (Mulu et al., 2015) | Title and Abstract |
| 1. (Gesesew et al., 2017) | Title and Abstract |
| 1. (Hassan et al., 2016) | Title and Abstract |
| 1. (Ali, 2012) | Title and Abstract |
| 1. (John et al., 2020) | Title and Abstract |
| 1. (Medhin et al., 2021) | Title and Abstract |
| 1. (Tafess et al., 2018) | Title and Abstract |
| 1. (Nagu et al., 2017a) | Title and Abstract |
| 1. (Birlie et al., 2015) | Title and Abstract |
| 1. (Tolosie and Sharma, 2014) | Title and Abstract |
| 1. (Ismail and Bulgiba, 2013) | Title and Abstract |
| 1. (Tolosie and Sharma, 2014) | Title and Abstract |
| 1. (Nansera et al., 2012) | Title and Abstract |
| 1. (Burke et al., 2021) | Title and Abstract |
| 1. (Ermeko et al., 2021) | Title and Abstract |
| 1. (Misgina et al., 2019) | Title and Abstract |
| 1. (Abate et al., 2016) | Title and Abstract |
| 1. (Getaneh et al., 2022b) | Title and Abstract |
| 1. (Fiseha et al., 2015) | Title and Abstract |
| 1. (Alebel et al., 2018) | Title and Abstract |
| 1. (Ramirez et al., 2012) | Title and Abstract |
| 1. (Nansera et al., 2012) | Title and Abstract |
| 1. (Anye et al., 2020) | Title and Abstract |
| 1. (Ajari and Adewale, 2021) | Title and Abstract |
| 1. (Aung et al., 2019) | Title and Abstract |
| 1. (Teshome Kefale and Anagaw, 2017) | Title and Abstract |
| 1. (Wali et al., 2021) | Title and Abstract |
| 1. (Liu et al., 2021) | Title and Abstract |
| 1. (Shah et al., 2021) | Title and Abstract |
| 1. (Akombi et al., 2017) | Title and Abstract |
| 1. (Akombi et al., 2017) | Title and Abstract |
| 1. (Worku et al., 2020) | Title and Abstract |
| 1. (Chowdhury et al., 2020) | Title and Abstract |
| 1. (Bizuneh et al., 2024a) | Title and Abstract |
| 1. (Mekonnen, 2021) | Title and Abstract |
| 1. (Yakob et al., 2018) | Title and Abstract |
| 1. (Abebe and Angamo, 2015) | Title and Abstract |
| 1. (Bristedt et al., 2024) | Title and Abstract |
| 1. (Otiende et al., 2019) | Title and Abstract |
| 1. (Liao et al., 2024) | Title and Abstract |
| 1. (Ali et al., 2016) | Title and Abstract |
| 1. (YAZEW and BEKELE, 2021) | Title and Abstract |
| 1. (Rose et al., 2014a) | Title and Abstract |
| 1. (Ladomenou et al., 2010) | Title and Abstract |
| 1. (Kerac et al., 2011) | Title and Abstract |
| 1. (Caminero and Scardigli, 2016) | Title and Abstract |
| 1. (Martorell, 2017) | Title and Abstract |
| 1. (Weiser et al., 2011) | Title and Abstract |
| 1. (Ifebunandu et al., 2012) | Title and Abstract |
| 1. (Au-Yeung et al., 2011) | Title and Abstract |
| 1. (Ifa, 2018) | Title and Abstract |
| 1. (Timilsina and Regmi, 2014) | Title and Abstract |
| 1. (Adamu et al., 2017) | Title and Abstract |
| 1. (Alemayehu et al., 2014) | Title and Abstract |
| 1. (IIiyasu et al., 2016) | Title and Abstract |
| 1. (Bwembya et al., 2024) | Title and Abstract |
| 1. (Ugwu et al., 2021) | Title and Abstract |
| 1. (Dare, 2016) | Title and Abstract |
| 1. (Mekuria and Debelo, 2018) | Title and Abstract |
| 1. (Worede and Abitew, 2021) | Title and Abstract |
| 1. (Musa et al., 2015) | Title and Abstract |
| 1. (Sullivan and Nathavitharana, 2022) | Title and Abstract |
| 1. (Monepya, 2022) | Title and Abstract |
| 1. (Kraef et al., 2021) | Title and Abstract |
| 1. (Ramosoeu, 2021) | Title and Abstract |
| 1. (Tachbele et al., 2017) | Title and Abstract |
| 1. (Temitayo-Oboh et al., 2022a) | Title and Abstract |
| 1. (Aderaye, 2007) | Title and Abstract |
| 1. (Mesfin et al., 2018) | Title and Abstract |
| 1. (Shipanga, 2019) | Title and Abstract |
| 1. (Ramadhani, 2017) | Title and Abstract |
| 1. (Molie et al., 2019) | Title and Abstract |
| 1. (Ekemu, 2017) | Title and Abstract |
| 1. (Tefera et al., 2019) | Title and Abstract |
| 1. (Mesfin et al., 2014) | Title and Abstract |
| 1. (Ambaw et al., 2015) | Title and Abstract |
| 1. (Amede et al., 2020) | Title and Abstract |
| 1. (Tamuzi et al., 2021) | Title and Abstract |
| 1. (Adeiza et al., 2014) | Title and Abstract |
| 1. (Haile et al., 2023) | Title and Abstract |
| 1. (Semunigus et al., 2016) | Title and Abstract |
| 1. (Semunigus et al., 2016) | Title and Abstract |
| 1. (Kassu et al., 2008) | Title and Abstract |
| 1. (Demissie and Belayneh, 2021) | Title and Abstract |
| 1. (Ayalaw et al., 2015b) | Title and Abstract |
| 1. (Aljohaney, 2018) | Title and Abstract |
| 1. (Tesema et al., 2020) | Title and Abstract |
| 1. (Ajema et al., 2020) | Title and Abstract |
| 1. (Ekemu, 2023) | Title and Abstract |
| 1. (Trinh et al., 2015) | Title and Abstract |
| 1. (Aminu, 2019) | Title and Abstract |
| 1. (Sifer and Getachew, 2024) | Title and Abstract |
| 1. (Tesema et al., 2019) | Title and Abstract |
| 1. (DEMSEW, 2019) | Title and Abstract |
| 1. (Kassa et al., 2013) | Title and Abstract |
| 1. (Hu et al., 2022) | Title and Abstract |
| 1. (Tshitenge et al., 2018) | Title and Abstract |
| 1. (Ugwu et al., 2021) | Title and Abstract |
| 1. (Mulu et al., 2015) | Title and Abstract |
| 1. (Shilongo, 2022) | Title and Abstract |
| 1. (Sade, 2013) | Title and Abstract |
| 1. (Ramosoeu, 2021) | Title and Abstract |
| 1. (Ramadhani, 2017) | Title and Abstract |
| 1. (Monepya, 2022) | Title and Abstract |
| 1. (Medhin et al., 2021) | Title and Abstract |
| 1. (Mawuta, 2017) | Title and Abstract |
| 1. (Lonsako, 2017) | Title and Abstract |
| 1. (Kitiabi and Cranmer, 2020) | Title and Abstract |
| 1. (Kidanemariam, 2014) | Title and Abstract |
| 1. (Hosu et al., 2024) | Title and Abstract |
| 1. (Hamusse, 2017) | Title and Abstract |
| 1. (Gyamfi–Gyimah, 2019) | Title and Abstract |
| 1. (Gondar, 2015) | Title and Abstract |
| 1. (Gibson, 2021) | Title and Abstract |
| 1. (Ekemu, 2017) | Title and Abstract |
| 1. (Afferu et al., 2020) | Title and Abstract |
| 1. (Aderaye, 2007) | Title and Abstract |
| 1. (Bukundi, 2020) | Title and Abstract |
| 1. (Medhin et al., 2021) | Title and Abstract |
| 1. (Mitku et al., 2016) | Title and Abstract |
| 1. (Desta et al., 2021) | Title and Abstract |
| 1. (Suara and Aryee, 2018) | Title and Abstract |
| 1. (Muhie, 2024) | Title and Abstract |
| 1. (Tsegaye et al., 2023a) | Title and Abstract |
| 1. (Mera et al., 2020) | Title and Abstract |
| 1. (Musa et al., 2015) | Title and Abstract |
| 1. (Kebede et al., 2021d) | Title and Abstract |
| 1. (Tachbele et al., 2017) | Title and Abstract |
| 1. (Jabir et al., 2022b) | Title and Abstract |
| 1. (Assebe et al., 2015b) | Title and Abstract |
| 1. (HAWULET, 2021) | Title and Abstract |
| 1. (Bizuneh et al., 2024b) | Title and Abstract |
| 1. (Rocha et al., 2021) | Title and Abstract |
| 1. (Preidis et al., 2011) | Title and Abstract |
| 1. (Marie et al., 2022) | Title and Abstract |
| 1. (Tanue et al., 2019) | Title and Abstract |
| 1. (Tachbele et al., 2017) | Title and Abstract |
| 1. (Ndirangu et al., 2011) | Title and Abstract |
| 1. (Reepalu et al., 2016) | Title and Abstract |
| 1. (Otiende et al., 2019) | Title and Abstract |
| 1. (Geleso, 2020) | Title and Abstract |
| 1. (Teshale et al., 2021) | Title and Abstract |
| 1. (Demissie and Belayneh, 2021) | Title and Abstract |
| 1. (Biyazin et al., 2022) | Title and Abstract |
| 1. (AHMED, 2022) | Title and Abstract |
| 1. (Kiros et al., 2022) | Title and Abstract |
| 1. (Toru et al., 2022) | Title and Abstract |
| 1. (Asuke et al., 2020) | Title and Abstract |
| 1. (Zemariam et al., 2024b) | Title and Abstract |
| 1. (Dodd et al., 2017) | Title and Abstract |
| 1. (Bristedt et al., 2024) | Title and Abstract |
| 1. (Worede and Abitew, 2021) | Title and Abstract |
| 1. (Mandalakas et al., 2020) | Title and Abstract |
| 1. (Getaneh et al., 2022b) | Title and Abstract |
| 1. (Peters et al., 2019) | Title and Abstract |
| 1. (Jerene et al., 2006) | Title and Abstract |
| 1. (Akombi et al., 2017) | Title and Abstract |
| 1. (Ramirez et al., 2012) | Title and Abstract |
| 1. (Seyoum et al., 2022) | Title and Abstract |
| 1. (Kegne et al., 2024) | Title and Abstract |
| 1. (Birhanu et al., 2024) | Title and Abstract |
| 1. (Debash et al., 2023) | Title and Abstract |
| 1. (Jabir et al., 2022a) | Title and Abstract |
| 1. (Abdilahi et al., 2024) | Title and Abstract |
| 1. (Duru et al., 2014) | Title and Abstract |
| 1. (Mekebo et al., 2020) | Title and Abstract |
| 1. (Ajari and Adewale, 2021) | Title and Abstract |
| 1. (Gisso et al., 2022) | Title and Abstract |
| 1. (Chowdhury et al., 2020) | Title and Abstract |
| 1. (Kebede Bizuneh et al., 2024) | Title and Abstract |
| 1. (Bizuneh et al., 2024b) | Title and Abstract |
| 1. (Tiruneh and Deyas, 2020) | Title and Abstract |
| 1. (Mequanente et al., 2022) | Title and Abstract |
| 1. (Tadege, 2018) | Title and Abstract |
| 1. (Palme et al., 2001b) | Title and Abstract |
| 1. (Liu et al., 2015) | Title and Abstract |
| 1. (Assefa et al., 2022) | Title and Abstract |
| 1. (Said et al., 2017) | Title and Abstract |
| 1. (Mamo et al., 2021) | Title and Abstract |
| 1. (Merid et al., 2019) | Title and Abstract |
| 1. (Abuogi et al., 2013) | Title and Abstract |
| 1. (Anye et al., 2020) | Title and Abstract |
| 1. (Enju et al., 2015) | Title and Abstract |
| 1. (Alebel et al., 2020a) | Title and Abstract |
| 1. (Zeleke, 2016) | Title and Abstract |
| 1. (Palme et al., 2001a) | Title and Abstract |
| 1. (Shasho et al., 2024) | Title and Abstract |
| 1. (Venturini et al., 2014) | Title and Abstract |
| 1. (Mandalakas et al., 2020) | Title and Abstract |
| 1. (Li et al., 2013) | Title and Abstract |
| 1. (Fry et al., 2019) | Title and Abstract |
| 1. (Martinson et al., 2009) | Title and Abstract |
| 1. (Krauss et al., 2015) | Title and Abstract |
| 1. (Hesseling et al., 2005) | Title and Abstract |
| 1. (Marais et al., 2007) | Title and Abstract |
| 1. (Zunza et al., 2017) | Title and Abstract |
| 1. (Cohen et al., 2008) | Title and Abstract |
| 1. (Edmonds et al., 2009) | Title and Abstract |
| 1. (Marcy et al., 2016) | Title and Abstract |
| 1. (Schaaf et al., 2007) | Title and Abstract |
| 1. (Mandalakas et al., 2008) | Title and Abstract |
| 1. (Cruz and Starke, 2007) | Title and Abstract |
| 1. (Naidoo et al., 2017) | Title and Abstract |
| 1. (Bong et al., 2007) | Title and Abstract |
| 1. (Githinji et al., 2018) | Title and Abstract |
| 1. (Melkamu et al., 2020) | Title and Abstract |
| 1. (Zar, 2008) | Title and Abstract |
| 1. (Patel et al., 2013) | Title and Abstract |
| 1. (Fairlie et al., 2014) | Title and Abstract |
| 1. (Feldacker et al., 2012) | Title and Abstract |
| 1. (López-Varela et al., 2015) | Title and Abstract |
| 1. (Jacobs et al., 2020) | Title and Abstract |
| 1. (Zar et al., 2007) | Title and Abstract |
| 1. (Sanogo et al., 2020) | Title and Abstract |
| 1. (Isaakidis et al., 2015) | Title and Abstract |
| 1. (Sánchez-Albisua et al., 2002) | Title and Abstract |
| 1. (Hall et al., 2017) | Title and Abstract |
| 1. (Aggerbeck et al., 2018) | Title and Abstract |
| 1. (Zar et al., 2010) | Title and Abstract |
| 1. (Rose et al., 2014b) | Title and Abstract |
| 1. (Elenga et al., 2005) | Title and Abstract |
| 1. (Dangor et al., 2013) | Title and Abstract |
| 1. (Sanjeeva et al., 2013) | Title and Abstract |
| 1. (Onyango et al., 2018) | Title and Abstract |
| 1. (Swaminathan and Rekha, 2010) | Title and Abstract |
| 1. (Palme et al., 2001b) | Title and Abstract |
| 1. (Naidoo et al., 2022) | Title and Abstract |
| 1. (Jabir et al., 2022b) | Title and Abstract |
| 1. (Zunza et al., 2017) | Title and Abstract |
| 1. (Ambaw et al., 2015) | Title and Abstract |
| 1. (Frigati et al., 2018) | Title and Abstract |
| 1. (Tamuzi et al., 2021) | Title and Abstract |
