# Supplementary figures and images for: Burden of mortality and its predictors among TB-HIV co-infected patients in Ethiopia: Systematic review and meta-analysis
Source: PLoS One. 2024 Nov 7;19(11):e0312698. doi: 10.1371/journal.pone.0312698 (PMC11542784; doi:10.1371/journal.pone.0312698)

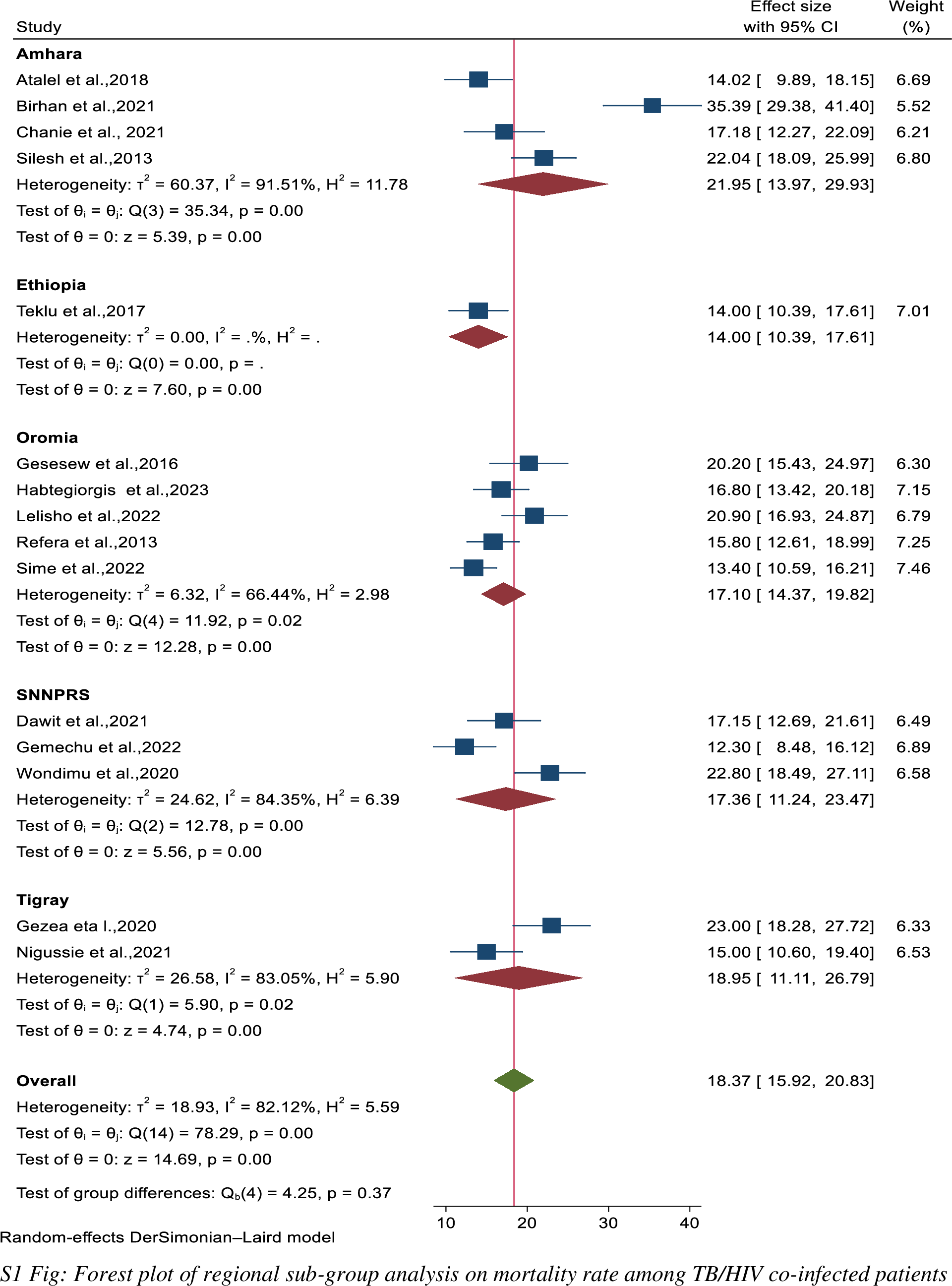

Supplement: S1 Fig — (TIF) [file pone.0312698.s006.tif]

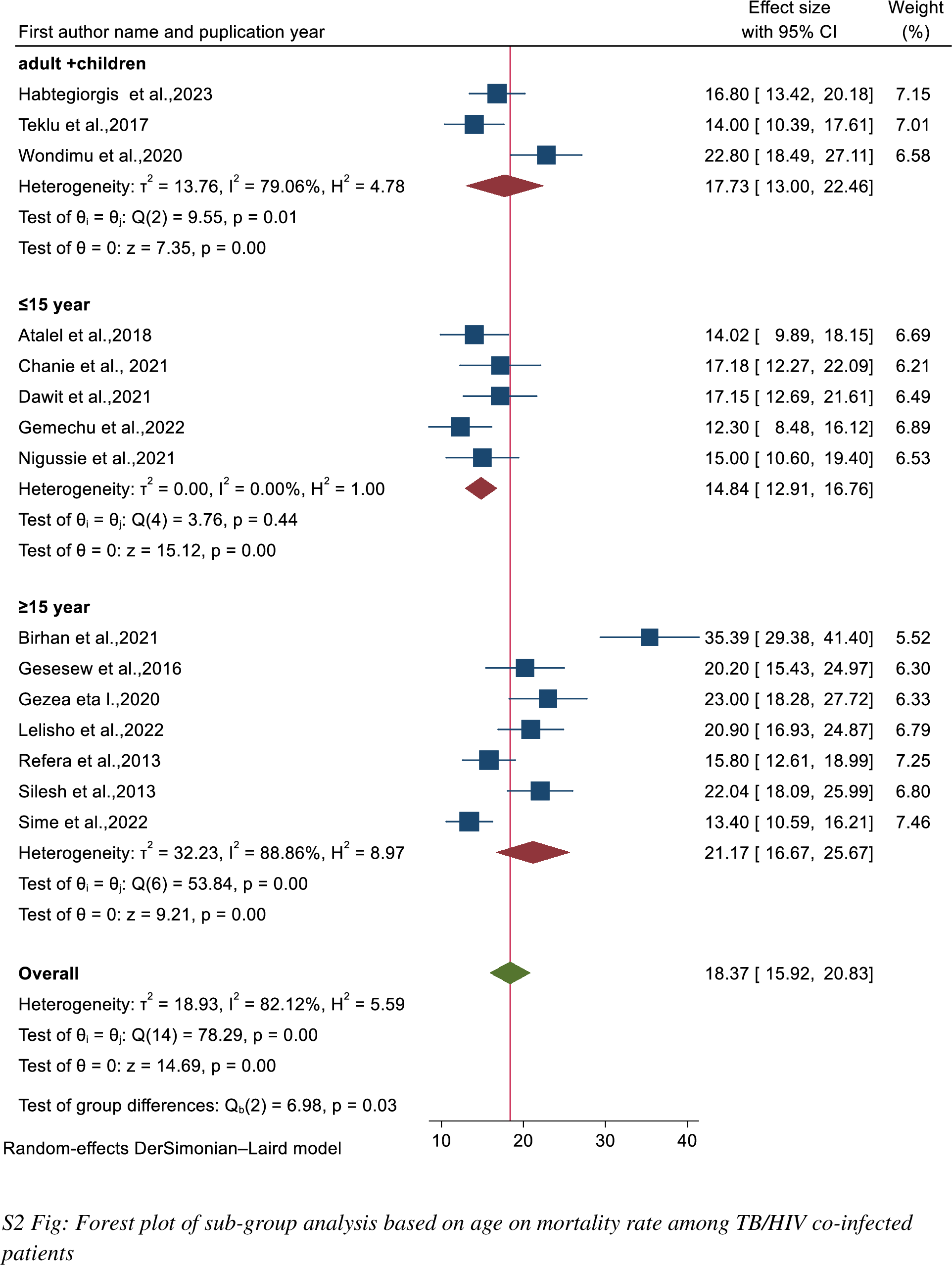

Supplement: S2 Fig — (TIF) [file pone.0312698.s007.tif]

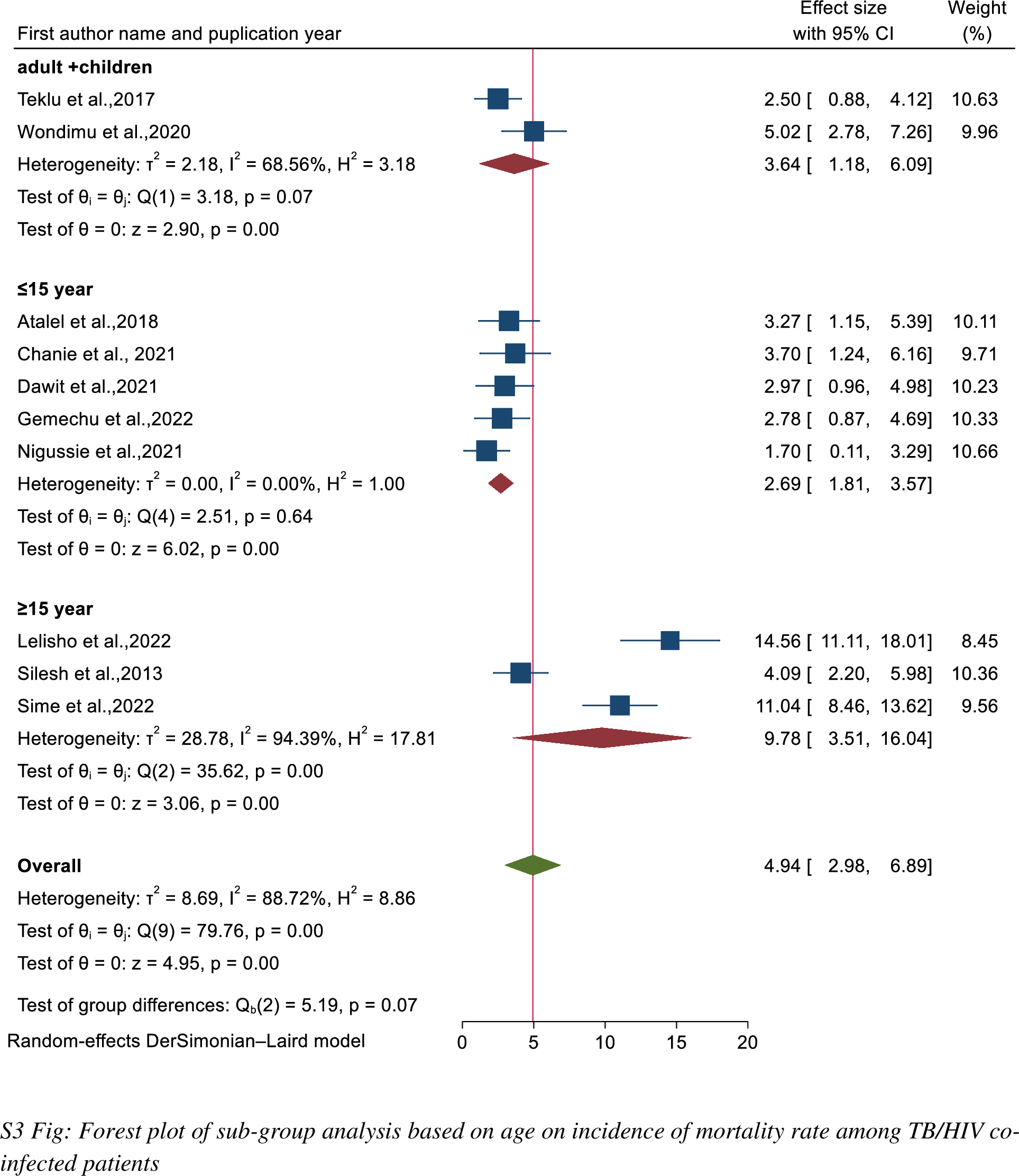

Supplement: S3 Fig — (TIF) [file pone.0312698.s008.tif]

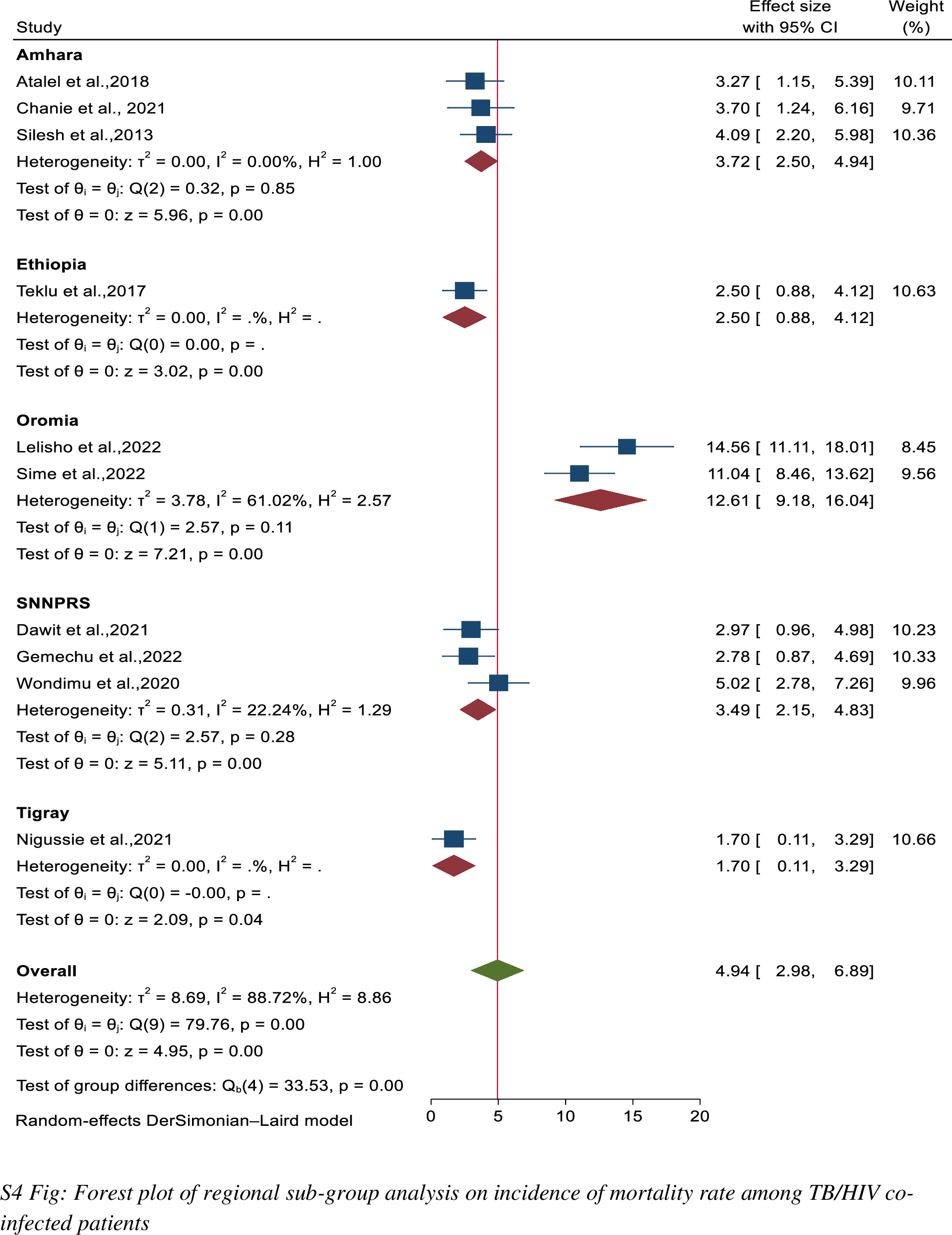

Supplement: S4 Fig — (TIF) [file pone.0312698.s009.tif]

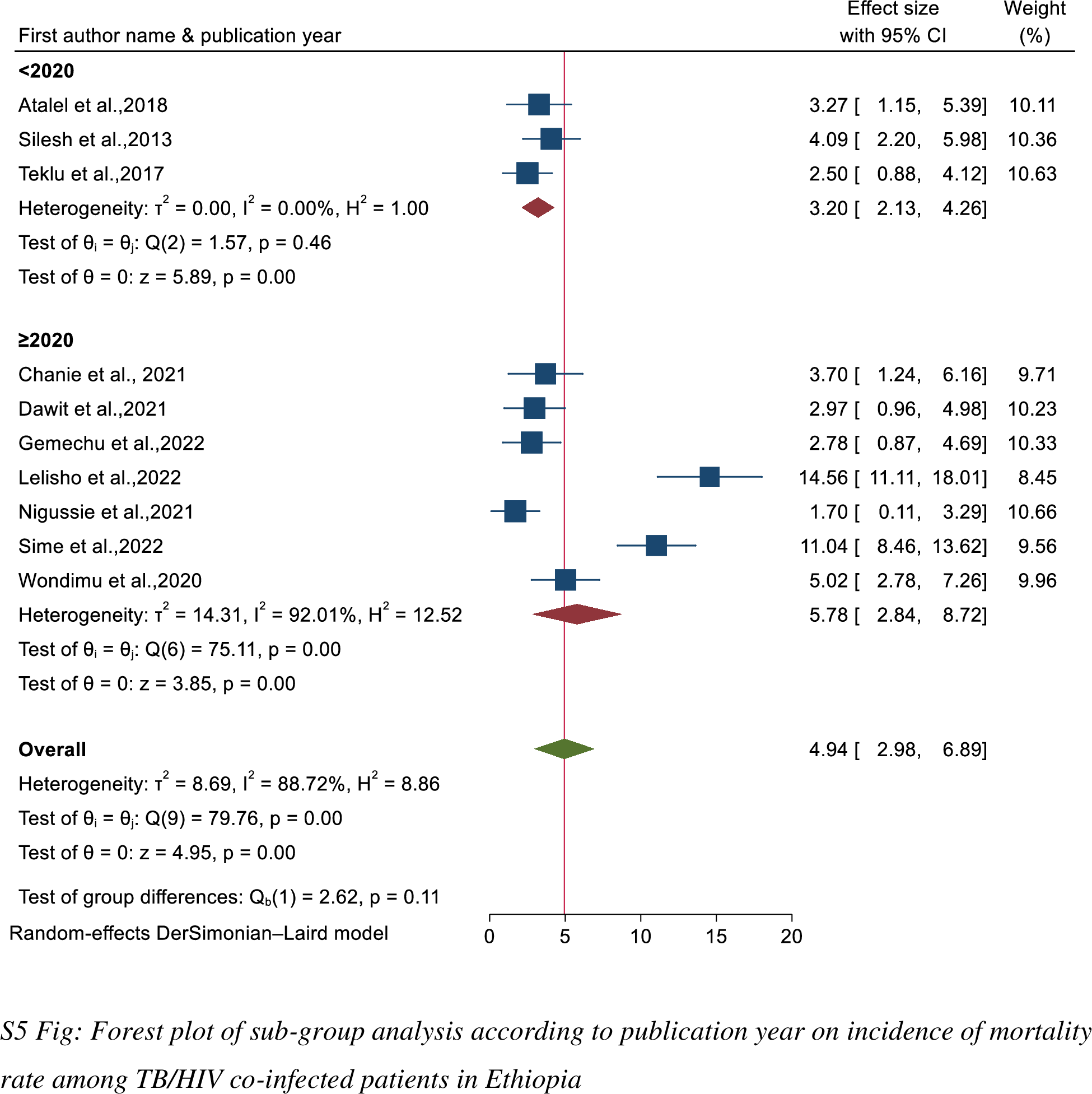

Supplement: S5 Fig — (TIF) [file pone.0312698.s010.tif]

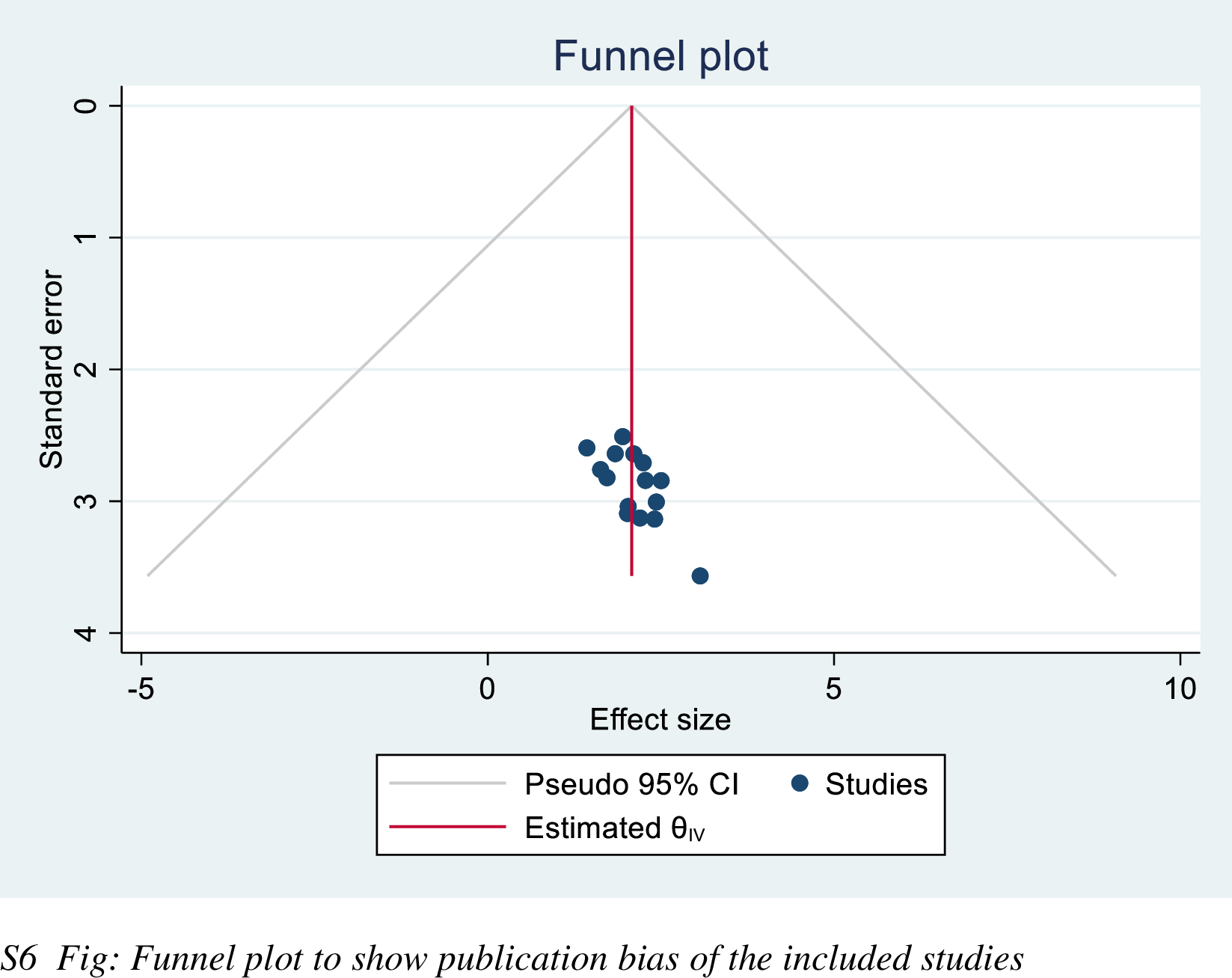

Supplement: S6 Fig — (TIF) [file pone.0312698.s011.tif]

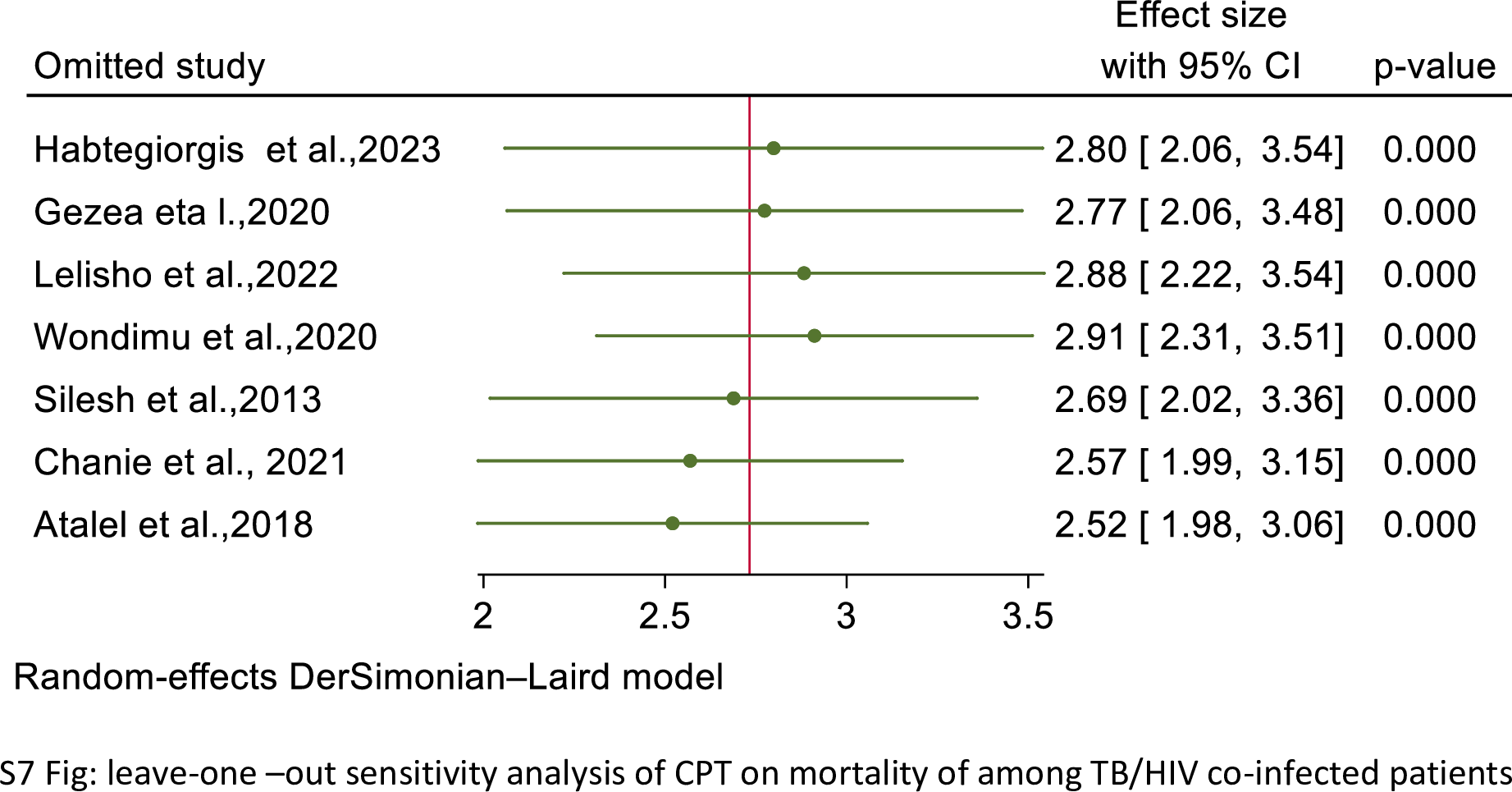

Supplement: S7 Fig — (TIF) [file pone.0312698.s012.tif]

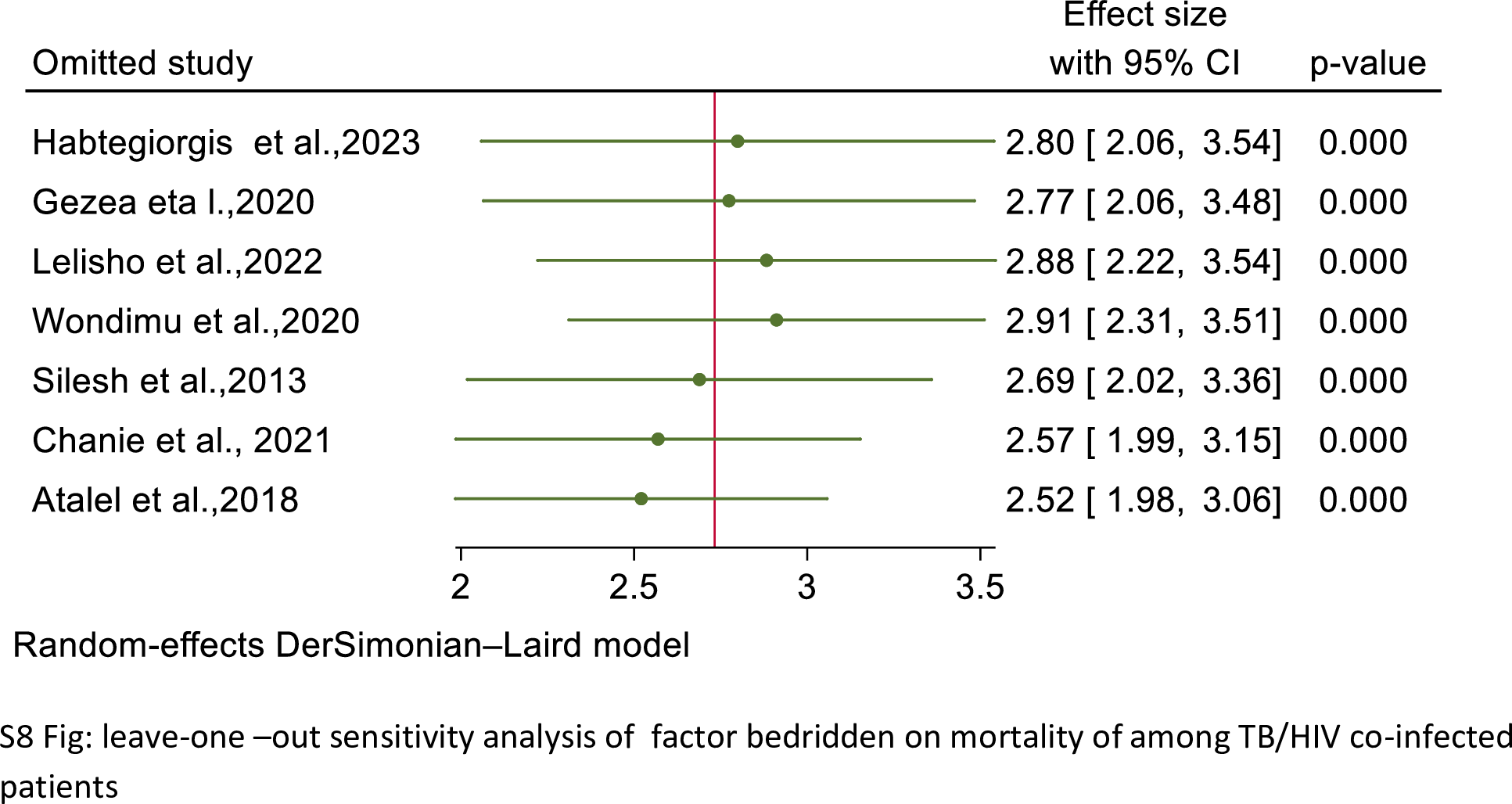

Supplement: S8 Fig — (TIF) [file pone.0312698.s013.tif]

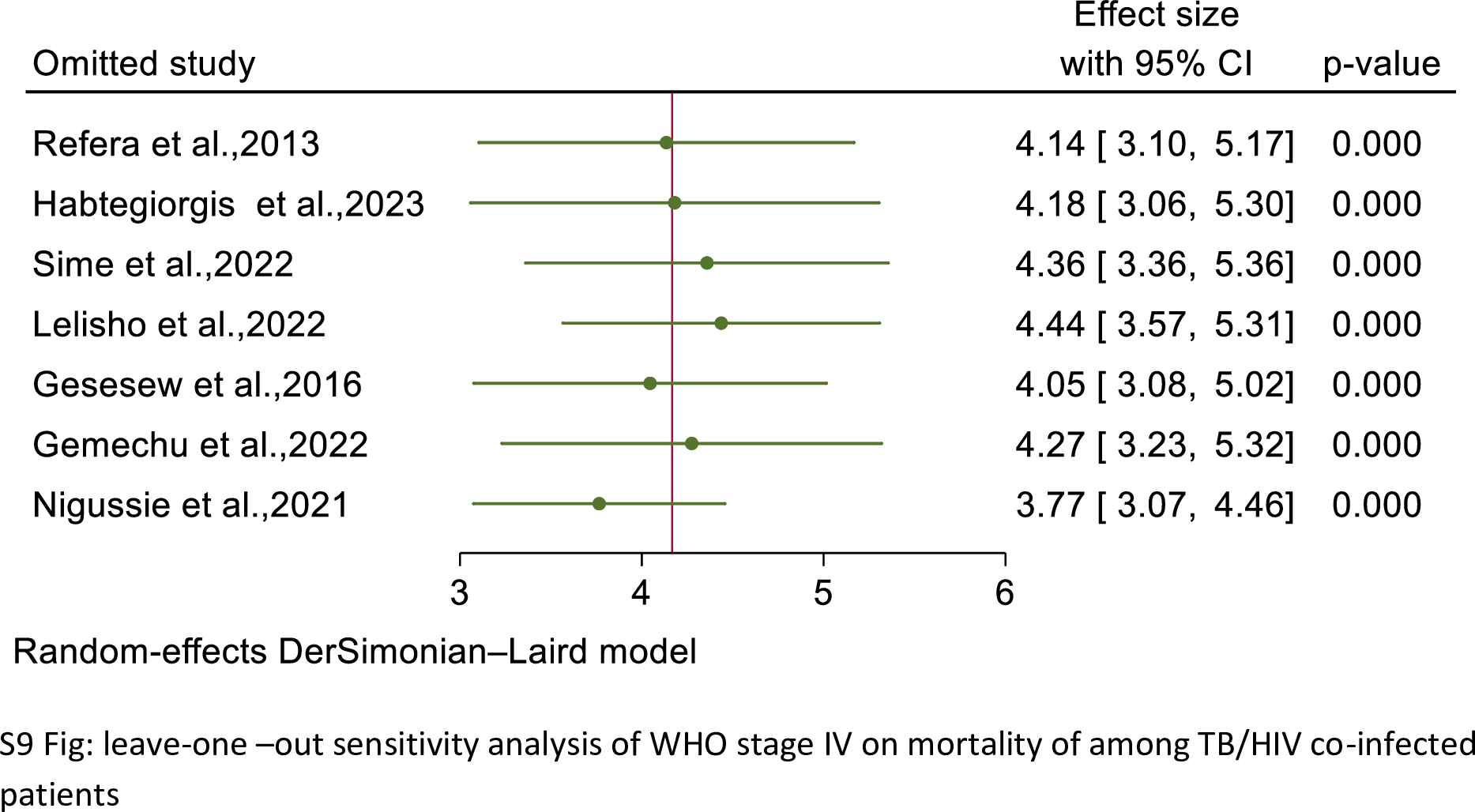

Supplement: S9 Fig — (TIF) [file pone.0312698.s014.tif]

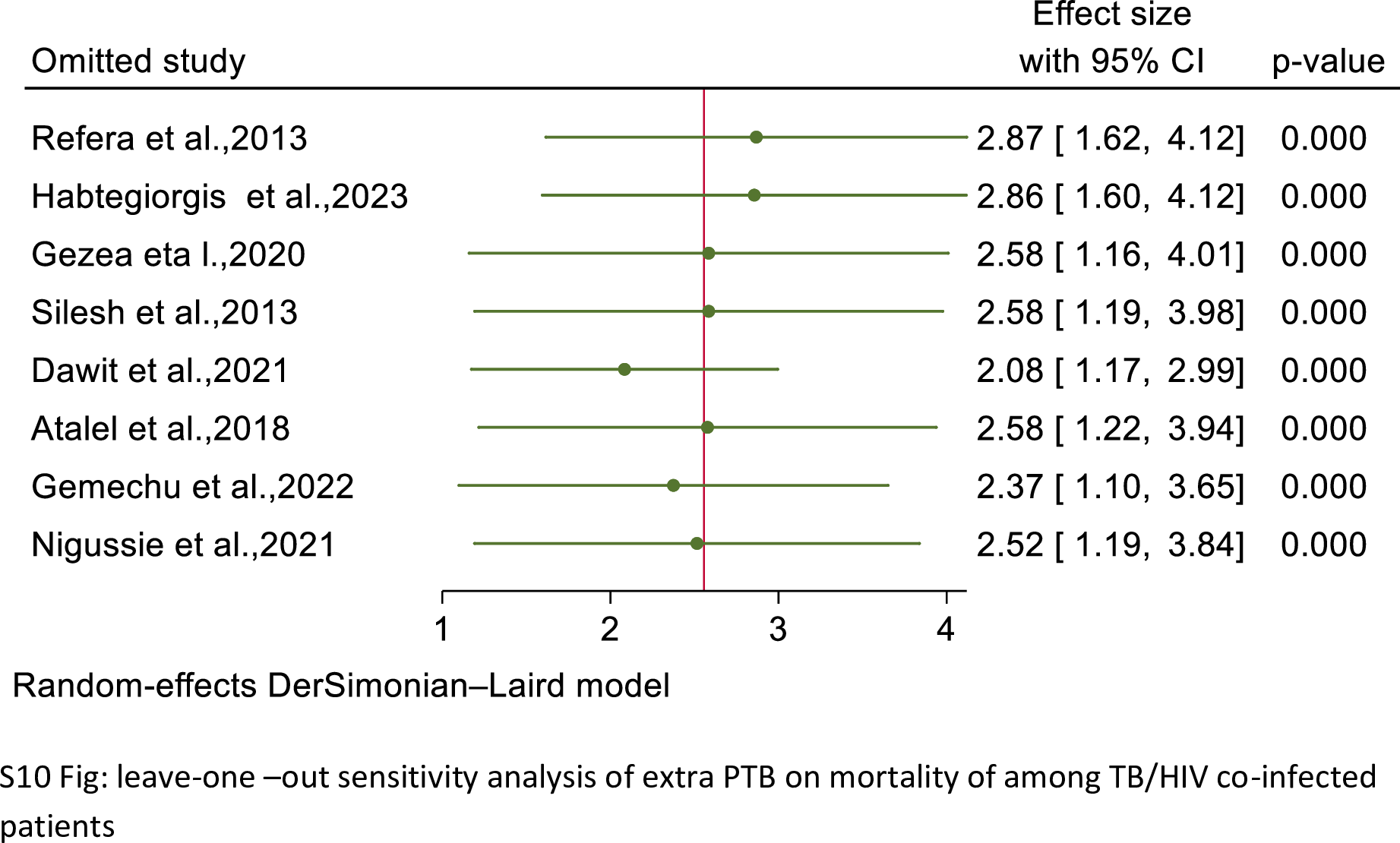

Supplement: S10 Fig — (TIF) [file pone.0312698.s015.tif]
