# Supplementary material for: Burden of mortality and its predictors among TB-HIV co-infected patients in Ethiopia: Systematic review and meta-analysis
Source: PLoS One. 2024 Nov 7;19(11):e0312698. doi: 10.1371/journal.pone.0312698 (PMC11542784; doi:10.1371/journal.pone.0312698)
Supplement: Attachment — Submitted filename: Response to reviewers.pdf [file pone.0312698.s016.pdf]

Response to Reviewers and editor

Point by point responses

We would like to take this opportunity to thank the reviewers and editor for sharing their view and constructive comments. The comments were very important which further improves the quality of our manuscript. The point-by-point responses for each of the comments are provided in the following pages. **Our responses are written in blue font color.**

Journal requirements

1. Please ensure that your manuscript meets PLOS ONE's style requirements, including those for file naming

Authors' Response

We are grateful to this comment of technical relevance. Thus, we have ensured that our manuscript meets PLOS ONE's style requirements, including those for file naming

2. We note that there is identifying data in the Supporting Information file <S1Table.docx>. Due to the inclusion of these potentially identifying data, we have removed this file from your file inventory

Authors' Response

We accept the comment and remove all personal information (Search date and location) and re-upload the data.

3. As required by our policy on Data Availability, please ensure your manuscript or supplementary information includes the following:

   A numbered table of all studies identified in the literature search, including those that were excluded from the analyses. For every excluded study, the table should list the reason(s) for exclusion.

   A table of all data extracted from the primary research sources for the systematic review and/or meta-analysis. The table must include the following information for each study:

   Name of data extractors and date of data extraction

   Confirmation that the study was eligible to be included in the review

   All data extracted from each study for the reported systematic review and/or meta-analysis that would be needed to replicate your analyses.

   An explanation of how missing data were handled.

This information can be included in the main text, supplementary information, or relevant data repository. Please note that providing these underlying data is a requirement for publication in this journal

Authors' Response

Thank you for this concern. We clearly put this concern in figure one that showed all studies identified in the literature search including those that were excluded from the analyses with reasons of exclusion.

Accepting the comment, we have put the extracted data on (S1 data) of supporting information on the revised manuscript. The date and the names of the data extractors were include on the main document of the revised version of this manuscript. The eligibility of the include studies were assessed on the quality assessment part of methods section.

As detailed in the methods sections, the pooled mortality rate among TB- HIV co-infected patients and its predictors was estimated using random effects model using DerSimonian-Laird model weight by utilizing Stata 17 statistical software.

Regarding to how missing data were handled, an explanation is done on the revised manuscript (page 6).

4. Please review your reference list to ensure that it is complete and correct. If you have cited papers that have been retracted, please include the rationale for doing so in the manuscript text, or remove these references and replace them with relevant current references. Any changes to the reference list should be mentioned in the rebuttal letter that accompanies your revised manuscript. If you need to cite a retracted article, indicate the article's retracted status in the References list and also include a citation and full reference for the retraction notice

Authors' Response

The authors are very grateful of these constructive comments. After read the reference lists carefully, we made some correction and addressed the incomplete one. We are sure that we have not cited retracted references, almost all references are recent.

Response to Reviewer #1 comments

5. Some minor error in the construction of paragraphs. The author must revise some repetitions for example 2024. As a systematic review and meta-analysis I think that all the parts of this types of study has been completed…..the aims of the paper have been reached the results

reports or are in agreement with the conclusions of the study. It is the first systematic review of its kind done in the country. Should take this in consideration in doing a final assessment of its suitability for acceptance.

Authors' Response

First of all, we really thank the reviewer for his constructive comments and suggestions. The authors have accepted the comments and corrected it in the revised version of the manuscript

Reviewer #2:

Minor comments

1. Acronyms - The authors can improve clarity by introducing the full term followed by its abbreviation when first mentioned, and then consistently using the acronym in subsequent references, rather than repeating the full term.

Authors' Response

Dear reviewer, we want to say thank you very much for your relevant, constructive and detail comments and suggestions that have a great value to improve our manuscript. The comment is taken and corrected accordingly.

2. To ensure consistent formatting, the authors should address some issues. In the discussion section, the words 'REMEMBER' is in capital letters and should be corrected to lowercase. Additionally, 'S7 Figure' is highlighted in red in the results section, which needs adjustment. Lastly, one of the lines in Figure 1 of the results section requires fixing for clarity

Authors' Response

Thank you and we corrected it.

Major comments

1. There appears to be a lack of consistency in the naming of the outcome measures. For instance, the methods section refers to the outcome measures as 'pooled mortality rate' and 'pooled incidence mortality rate,' while the results section uses the term 'incidence density mortality rate.' Additionally, the term 'pooled proportion' is used in the conclusion. It is important to ensure consistency in the terminology used for the outcome measures throughout the manuscript.

It would be helpful to include definitions for the two outcome measures in the methods section and to explain how they were calculated. This could involve specifying both the numerator and denominator for each outcome to ensure clarity and consistency.

Authors' Response

Based on the comment, correction is made on the revised version of the manuscript.

2. The manuscript would benefit from thorough proofreading and editing to enhance clarity and accuracy.

Authors' Response

Thank you for your constructive comments. After we have read carefully through the whole document, we properly addressed the concerned issues.

Accepting the comment, the authors have read thoroughly and edited carefully the whole manuscript before submission.

3. In the methods section of the abstract, authors may not need to mention the quality assessment of primary studies or publication bias; these details could be reserved for the methods section of the manuscript. Instead, the abstract should include the number of papers included in the systematic review and meta-analysis, the duration of the search period (from 2004 to 2024), and the total sample size.

Authors' Response

Accepting the comment, the authors have corrected accordingly.

4. Additionally, specify the analyses performed with the data extracted from the primary studies, including any subgroup analyses conducted by region.

Authors' Response

After we extracted the data from primary studies on Microsoft excel, the analysis was performed by using STATA 17 statistical software. Then, the pooled mortality rate among TB-HIV co-infected patients and its predictors was estimated using random effects model using DerSimonian-Laird model weight.

Sub-group analysis was also performed using publication year, region, sample size and age of patients from the extracted data and presented using frost plot.
